# Comparing the molecular evolution and recombination patterns of predominant PRRSV-2 lineages co-circulating in China

**DOI:** 10.3389/fmicb.2024.1398470

**Published:** 2024-04-26

**Authors:** Riteng Zhang, Hui Li, Honglin Xie, Xiaolan Hou, Lixuan Zhou, Aiqiao Cao, Basit Zeshan, Yefei Zhou, Xinglong Wang

**Affiliations:** ^1^College of Veterinary Medicine, Northwest A&F University, Yangling, Shaanxi, China; ^2^Shenzhen Institute of Quality and Safety Inspection and Research, Shenzhen, Guangdong, China; ^3^Faculty of Sustainable Agriculture, Universiti Malaysia Sabah, Sandakan, Sabah, Malaysia; ^4^Department of Life Science, Nanjing Xiaozhuang University, Nanjing, Jiangsu, China

**Keywords:** PRRSV, Bayesian, recombination hotspots, evolution dynamics, glycosylation

## Abstract

Porcine reproductive and respiratory syndrome virus (PRRSV) poses widespread epidemics in swine herds, yet the drivers underlying lineage replacements/fitness dynamics remain unclear. To delineate the evolutionary trajectories of PRRSV-2 lineages prevalent in China, we performed a comprehensive longitudinal phylodynamic analysis of 822 viral sequences spanning 1991–2022. The objectives encompassed evaluating lineage dynamics, genetic diversity, recombination patterns and glycosylation profiles. A significant shift in the dominance of PRRSV-2 sub-lineages has been observed over the past 3 decades, transitioning from sub-lineage 8.7 to sub-lineage 1.8, followed by extensive diversification. The analysis revealed discordant recombination patterns between the two dominant viral sub-lineages 1.8 and 8.7, underscoring that modular genetic exchanges contribute significantly to their evolutionary shaping. Additionally, a strong association was found between recombination breakpoint locations and transcriptional regulatory sequences (TRSs). Glycosylation patterns also demonstrated considerable variability across sub-lineages and temporally, providing evidence for immune-driven viral evolution. Furthermore, we quantified different evolutionary rates across sub-lineages, with sub-lineage 1.8 uniquely displaying the highest nucleotide substitution rates. Taken together, these findings provide refined insight into the evolutionary mechanisms underpinning cyclic shifts in dominance among regionally circulating PRRSV sub-lineages.

## 1 Introduction

Porcine reproductive and respiratory syndrome virus (PRRSV) has posed substantial economic losses to the global swine industry, yet effective management and control of PRRS remain a challenge (Du et al., 2017). PRRSV is a single-stranded positive-sense RNA virus, and its genome is approximately 15.4 kb in length, containing at least 11 partially overlapping open reading frames (ORFs) (Yan et al., 2022). Initial outbreaks of PRRS were reported simultaneously in North America (late 1980s, Lelystad virus) and Western Europe (1990s, VR-2332). Since its emergence, PRRSV variants have constantly evolved, presenting as two genetically and antigenically heterogeneous viral populations: Betaarterivirus suid 1 (PRRSV-1) and Betaarterivirus suid 2 (PRRSV-2) (Kuhn et al., 2016). Historically, outbreaks within China are mostly classified as PRRSV-2, despite sporadic cases caused by PRRSV-1 also being documented (Guo et al., 2018; Xu et al., 2023). Restriction fragment length polymorphism (RFLP) typing was proposed as a tool for early comparative virology studies to differentiate wild-type from Ingelvac MLV vaccine-like strains (Trevisan et al., 2021). An important limitation of RFLP typing is its inability to accurately determine the genetic relatedness of PRRSVs, despite its widespread use in North America (Yim-im et al., 2023). To this end, a genetic classification system utilizing established nucleotide percent cut-off values was proposed in 2010 (Shi et al., 2010). This classification system grouped PRRSV type 2 viruses into nine lineages based on phylogenetic relationships in the ORF5 region, generally with intra-lineage diversity levels below 11%.

Porcine reproductive and respiratory syndrome virus strains have a long evolutionary history and diverged early in their origin. Four co-circulating lineage strains have been documented in China, including lineages 1, 3, 5, and 8 (Jiang et al., 2020). The early BJ-4 isolate belonged to sub-lineage 5.1 and shared only 99.6% sequence homology with VR2332 (Guo et al., 2018). This similarity suggests that lineage 5 may be imported or derived from a vaccine strain infection originating in North America. Lineage 5 demonstrated relatively stable and limited epidemicity (Gao et al., 2017). Since the emergence of highly pathogenic PRRSV (HP-PRRSV) in 2006, the frequent inter-regional spread has led lineage 8 to become the dominant circulating strain in swine herds, causing substantial economic losses to domestic pig production (Han et al., 2017). The evolution of HP-PRRSV was considered the result of the gradual accumulation of mutations in intermediate strains, represented by HB-1(sh)/2002 and HB-2(sh)/2002 (Li et al., 2023). Unlike other lineages of PRRSV-2, lineage 1 strains possess global pandemic potential and harbor higher genetic variation (Paploski et al., 2019). Also known as the NADC30-like strain, sub-lineage 1.8 emerged *circa* 2013 and propagated throughout China (Zhou et al., 2015). The resulting epidemic escalated in severity. Sub-lineage 1.5, designated as the NADC34-like strain, was initially detected in China in 2017 (Zhang et al., 2018). Subsequent surveillance data revealed that sub-lineages 1.5 and 1.8 accounted for 64% of the positive clinical specimens in 2021, significantly exceeding prevalence levels displayed by other genetic lineages (Xu et al., 2022). Although commercially available vaccines have shown some efficacy in reducing clinical symptoms, they have failed to prevent continued dissemination of lineage 1 strains. Lineage 3 isolates were originally restricted to Taiwan, after which sporadic distribution of this lineage occurred in Hong Kong (Sun et al., 2019). These viral populations have ultimately been classified into sub-lineages 3.1–3.4 (Zhang et al., 2019). Additionally, Sub-lineage 3.5 was another newly emerged variant circulating predominantly in southern China since 2010. Phylodynamic analyses stated that Taiwan was the origin of lineage 3 viruses (Sun et al., 2019).

Genetic mutation and recombination have been widely described as important drivers of PRRSV lineage evolution (Murtaugh et al., 2010). The calculated rate of PRRSV-2 nucleotide substitutions ranges from 8.7 × 10^−3^ to 1.1 × 10^−2^/site/year (Shi et al., 2010). Increasingly complex recombination landscapes have been reported in recent years (Yu et al., 2020). Strikingly, certain isolates such as FJLIUY-2017 and GXYN20220502 were observed to arise through recombination event between four sub-lineages (Liu et al., 2019). Holistically, recombination between dominant circulating sub-lineages in local swine populations may confer fitness advantages, resulting in variants more contagious or better at evading immune responses. However, key factors driving PRRSV multi-strain dynamics remain underexplored. To bridge these knowledge gaps, we conducted an updated phylogenetic and phylodynamic analysis, examining lineage movements, regional dynamics, ancestral origins, evolutionary rates, and recombination patterns in China over the past 3 decades. Synthesizing these insights will help uncover evolutionary forces shaping viral genetic diversity, which has implications for the design of potential antigen targets.

## 2 Materials and methods

### 2.1 Dataset compilation and initial sequence alignments

All publicly available PRRSV genome sequences originating from China until December 30, 2022, were retrieved from NCBI Genbank. Repeated sequences and culture-attenuated sequences were excluded, the final dataset consisted of 822 sequences. All sequences were aligned via MAFFT v7 with default parameters and trimmed in Geneious Prime 2022.2.2 software^1^ (Katoh et al., 2002). Additional details including accession number, isolate names, collection date, and geographical origin were documented in Supplementary Table S1. The *p*-distance analyses were calculated by MEGA v11 (Tamura et al., 2021).

To determine the insertion/deletion (indel) polymorphism patterns in NSP2, multiple sequence alignments were generated using Geneious Prime with VR-2332 set as the reference strain. All deletions and insertions were annotated, and the population size for each pattern was quantified.

### 2.2 Phylogenetic and evolutionary dynamic analysis

A Maximum Likelihood (ML) tree was constructed using IQ-TREE software under GTR + F + I + G4 nucleotide substitution model with ultrafast bootstraps (1,000 replicates) (Nguyen et al., 2015). Bayesian time-scaled trees based on the ORF5 gene were generated through a Markov chain Monte Carlo (MCMC) framework applied in BEAST v1.10.4 (Suchard et al., 2018). The lengths of the MCMC chain were set to 100 million generations and sampled every 10,000 steps. Convergence for the log file was assessed in Tracer v1.7.1, with all parameter effective sampling sizes >200 (Rambaut et al., 2018). Maximum clade credibility (MCC) trees for each run were summarized using TreeAnnotator v1.10.4 after discarding the first 10% as burn-in. All final trees were visualized using FigTree v1.4.4 annotated and plotted using the ggtree package.^2^ The root-to-tip genetic distances of virus strains and the time to the most recent common ancestor (tMRCA) were evaluated using TreeTime program (Sagulenko et al., 2018). Bayesian skyline plots (BSP) were constructed in Tracer to estimate the effective population size of diverse sub-lineages (Drummond et al., 2005).

### 2.3 Recombination detection

The viral data sets were preliminarily screened for recombination signals using seven algorithms implemented in the recombination detection program 5 (RDP5), including RDP, GENECONV, Bootscan, MaxChi, Chimera, SiScan, and 3seq. Only recombination events verified by at least four methods were further assessed for the possibility of recombination, with an acceptable cut-off *p* value lower than 0.05 (Martin et al., 2021). Putative recombinants were further verified by using the SimPlot v3.5.1 and VirusRecom program (Zhou et al., 2023). This multiple-pronged *in silico* approach conferred an enhanced degree of confidence in the identification of candidate sequences likely containing authentic recombination breakpoints. Following the aggregation of results from various analytical packages, major and minor parents along with breakpoint positions were integrated for downstream characterization. The terms “major” and “minor” parents are used to designate the parental isolates that contribute the larger and smaller fractions, respectively, based on genetic similarity.

### 2.4 N-glycosylation prediction and RFLP typing

To explore the temporal N-glycosylation patterns of PRRSV-2 strains in China, ORF5 sequences were submitted to the NetNGlyc web server for analysis.^3^ The default threshold of 0.5 was applied to predict potential N-glycosylation sites, whereas 0.75 and 0.9 threshold levels were then applied for additional stringency.

The RFLP typing currently used is based on three restriction enzyme (MluI, HincII, and SacII) cleavage sites within the ORF5 sequence, as referenced from the RFLP pattern archive maintained by the University of Minnesota (Trevisan et al., 2021). Statistical data analysis was performed using GraphPad Prism 9 software (GraphPad Software, Inc., La Jolla, CA, United States).

## 3 Results

### 3.1 Lineage classification and geographical distribution of PRRSV spread within China

**Figure 1:**
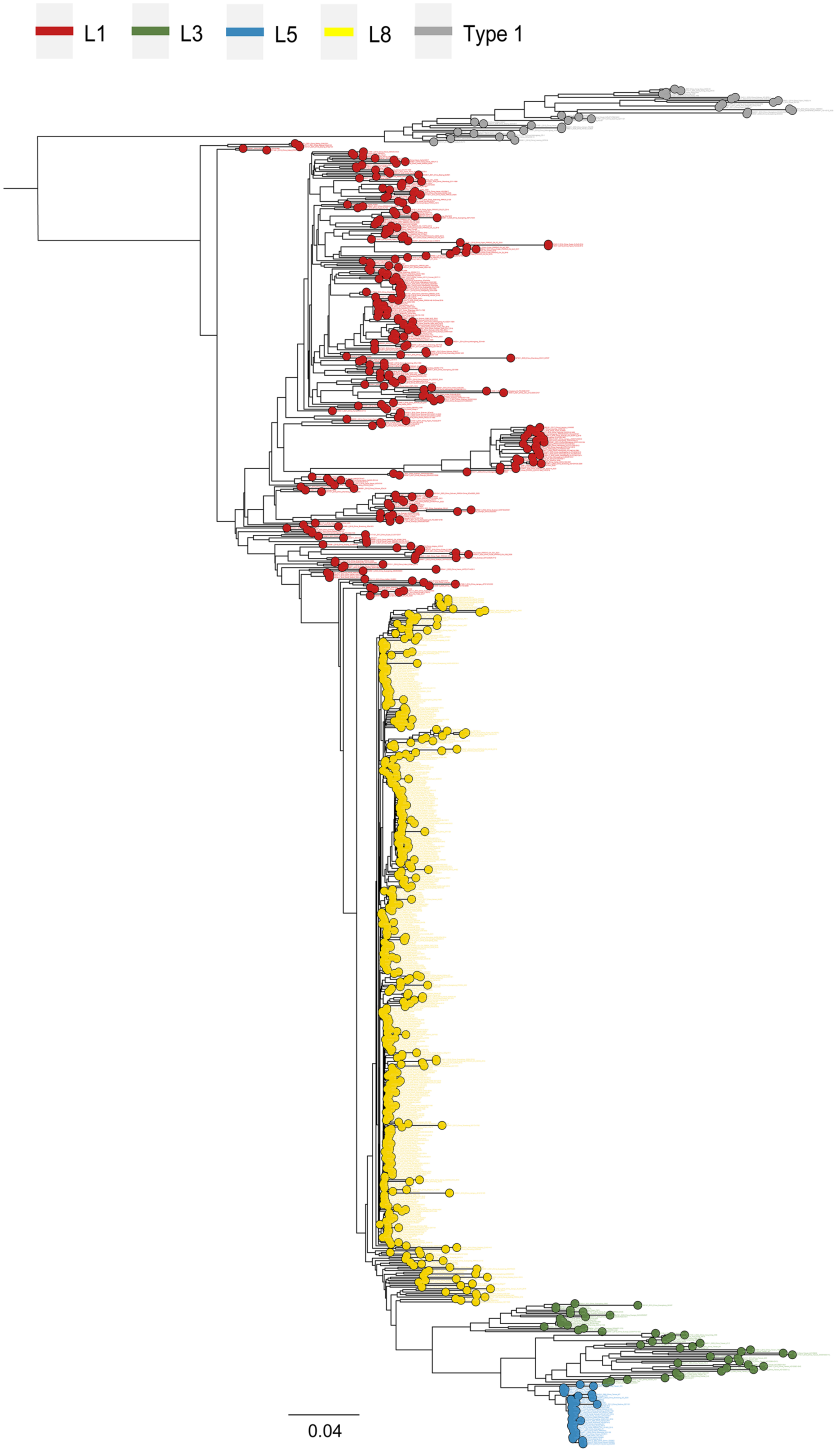
Maximum likelihood phylogeny reconstruction of 822 PRRSV whole-genome sequences. Sequences were analyzed using publicly available whole-genomes from China previously deposited in GenBank. Four main genetic lineages were distinguished by type 2 PRRSV strains: lineage 1, lineage 3, lineage 5, and lineage 8. Tip points and branches were presented in different colors according to lineages. Virus nomenclature followed a sequential order: GenBank accession number, collection date, geographic location, and strain name.

**Figure 2:**
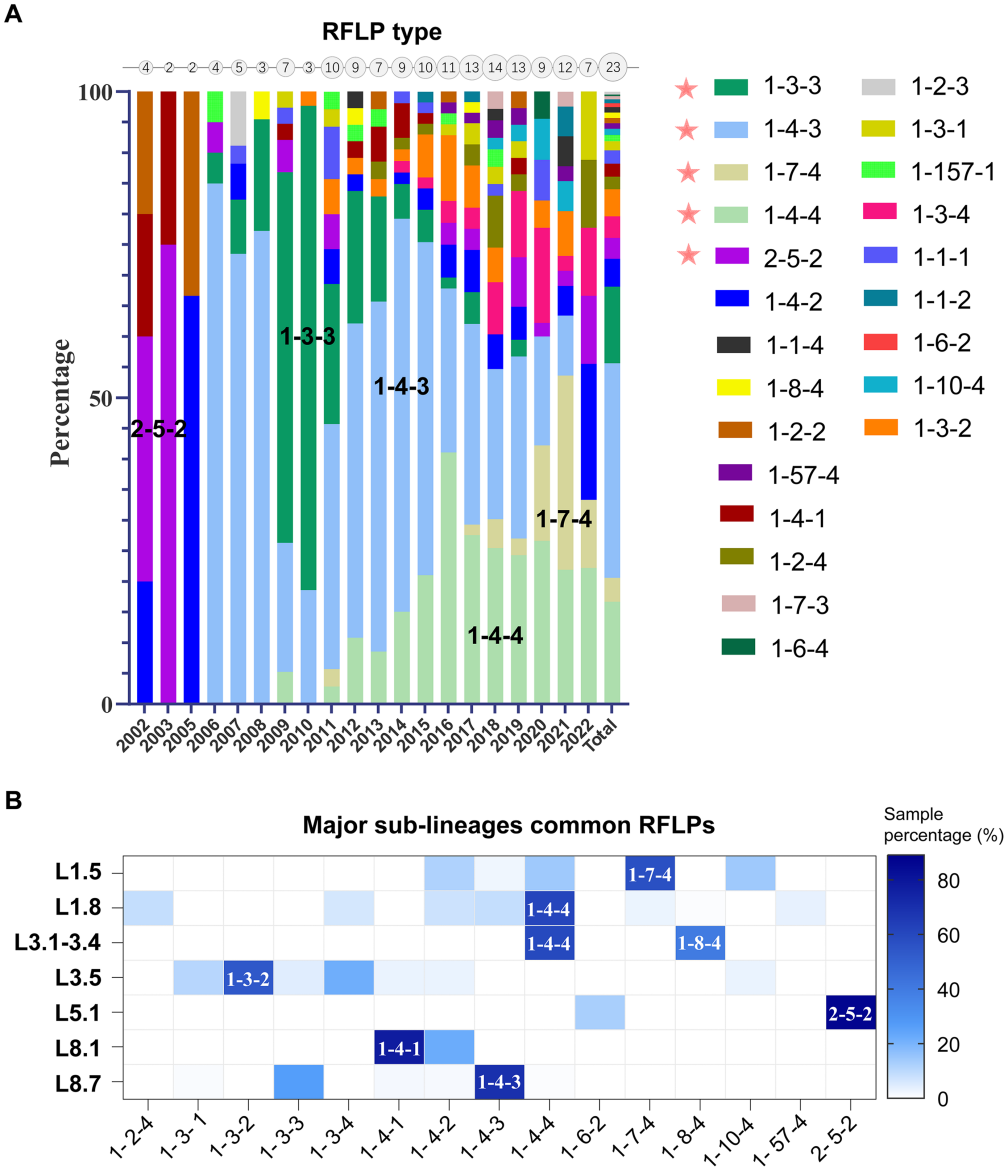
Genetic diversity of PRRSV-2 in mainland China as characterized by restriction fragment length polymorphism (RFLP) patterns. **(A)** Temporal analysis of RFLP pattern detection (Supported by ≥3 isolates). Circle sizes on the top axis denoted annual RFLP type counts, reflecting progressive expansion of new patterns. **(B)** Distribution of RFLP types across major phylogenetic clades, emphasizing the most prevalent RFLP patterns observed within each sub-lineage.

Phylogenetic lineage/sub-lineage assignment protocol incorporated 822 unique ID sequences, and the ML tree obtained by IQ-TREE confirmed the clustering pattern of the viruses into two major genotypes: PRRSV-1 (33 isolates) and PRRSV-2 (789 isolates) (Figure 1). Within type 2 PRRSVs, the demarcation into four established lineages: 1, 3, 5, and 8, persisted. Approximately 46.9% of the sequences were classified as lineage 8, and lineage 1 was next, comprising 32.7% (Supplementary Table S1). A notable diversification and further subdivision were observed specifically within lineage 1. Furthermore, a Bayesian phylogenetic tree, inferred from all ORF5 sequences, was depicted in Supplementary Figure S1A. Evolutionary divergence analysis indicated that the greatest genetic distance observed between lineages was 0.18287, occurring between sub-lineages 1.8 and 3.5 (Supplementary Figure S1B). The extent of PRRSV-2 genetic variation can also be observed from RFLP pattern perspective. Our study identified over 23 unique RFLP types (Supplementary Table S1). An analysis of RFLP detection trends revealed considerable temporal variations, with the accelerating enumeration of new RFLP patterns underscoring the extensive variability inherent in viral populations (Figure 2A). Additionally, common RFLP types for several significant sub-lineages were listed, such as sub-lineage 8.7 (1-4-3), sub-lineage 3.5 (1-3-2), sub-lineage 5.1 (2-5-2), sub-lineage 1.5 (1-7-4), and sub-lineage 1.8 (1-4-4) (Figure 2B).

**Figure 3:**
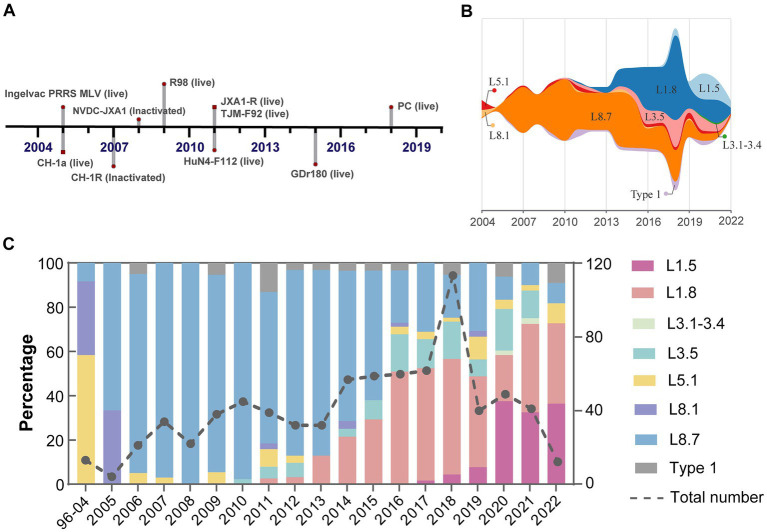
Shifts in dominance among PRRSV-2 sub-lineages in mainland China. **(A)** A timeline outlined the key milestone achievements of PRRSV vaccine development. **(B)** Temporal trends in PRRSV sub-lineage prevalence estimated using observation dates of all reported cases. **(C)** Relative frequency and quantity of different viral sub-lineages per year.

**Figure 4:**
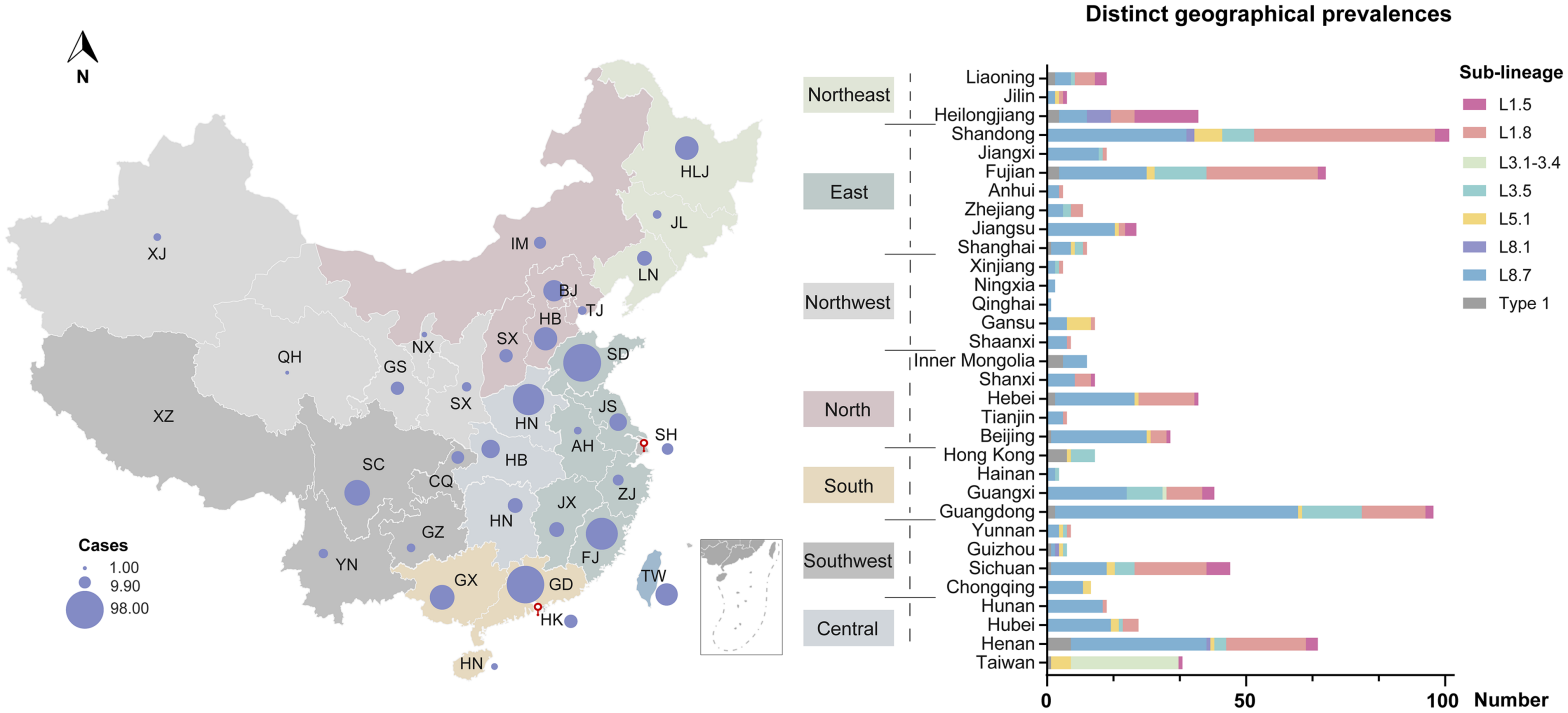
Geographical distribution of PRRSV-2 across different regions in China. Circle sizes represented the provincial isolate counts. Provinces were categorized into seven regions based on proximity—North, Northeast, East, Central, South, Southwest, and Northwest.

Despite widespread implementation of inactivated and modified live viral vaccines, viral genetic diversity persists in continued expansion (Figure 3A). Sub-lineage 8.7 has consistently dominated from 2005 through 2015 (Figure 3B). Notably, the prevalence of sub-lineages 1.8 and 1.5 increased markedly in 2016 and 2020, respectively, thereby supplanting sub-lineage 8.7 as the predominant variants circulating domestically (Figure 3C). In contrast, sub-lineages 5.1 and 3.5, along with type 1 isolates, maintained stability at low prevalence levels, with only minor fluctuations. Regional variations in the prevalence of different lineages/sub-lineages were also documented (Figure 4). Lineage 8 dominated in the North (63.5%) and Central (61.3%) regions in China, while lineage 1 dominated in the Northeast (55.2%). Lineage 3 reached its highest frequency in South China (20.8%).

Genetic diversity within the PRRSV virus population can be categorized based on NSP2 polymorphism. With increasing rounds of infection, the accumulated patterns of insertions/deletions within the viral genome elucidate its intricate evolutionary trajectories. Multiple sub-lineages have developed relatively stable and unique characteristics in the NSP2 replicase region and have been divided into 19 main indel patterns according to the amino acid framework (Supplementary Figure S2).

### 3.2 Evolutionary rate and population dynamic analyses

**Figure 5:**
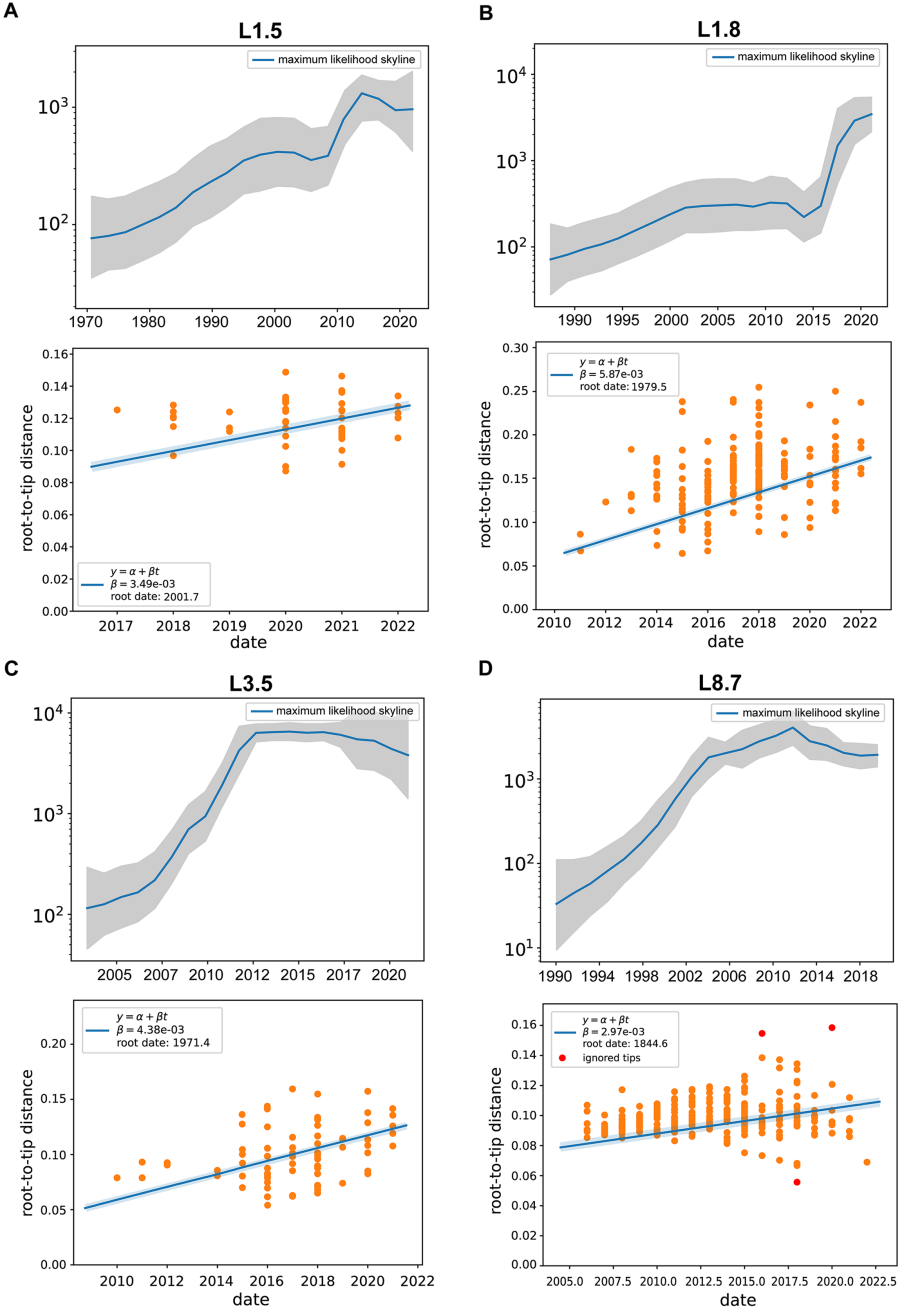
Evolutionary dynamics of different PRRSV-2 sub-lineages. **(A–D)** Effective population size (Ne) for PRRSV sub-lineages, with the middle blue line indicating median estimate and shaded grey area indicating the 95% confidence interval (CI). (Bottom) Root-to-tip distance plots on sampling dates and estimated evolutionary rates for PRRSV sub-lineages. Linear regressions were performed with Treetime, with yellow circles for data conforming to the linear model and red circles for outliers.

Differences in effective population sizes exist among PRRSV-2 sub-lineages, with fluctuating patterns of relative genetic diversity revealed under Bayesian skyline coalescent models (Figures 5A–D). Specifically, sub-lineage 8.7 experienced a consistent expansion initially, reaching its peak in 2012, followed by a gradual decline in population size and entering a plateau in 2018 (Figure 5D). For sub-lineage 1.8, the populations maintained relative stability between 2001 and 2011. Following a marginal decline after 2013, it embarked on a period of exponential growth (Figure 5B). The population size of sub-lineage 1.5 underwent rapid contraction beginning in 2014 and entered a plateau by 2019 (Figure 5A). By contrast, sub-lineage 3.5 achieved maximum lineage numbers prior to 2012, thereafter sustaining a steady state; post-2017, it began to shrink slowly (Figure 5C).

As shown in Supplementary Figure S3A, the mean evolutionary rate was estimated at 3.49 (95% HPD interval, 2.46–4.27) × 10^−3^ substitutions/site/year for sub-lineage 1.5 strains, 5.87 (95% HPD interval, 4.61–6.92) × 10^−3^ substitutions/site/year for sub-lineage 1.8 strains, and 4.38 (95% HPD interval, 3.54–5.73) × 10^−3^ substitutions/site/year for sub-lineage 3.5 strains, and 2.97 (95% HPD interval, 1.86–3.52) × 10^−3^ substitutions/site/year for sub-lineage 8.7 strains. These data indicated that sub-lineage 1.8 evolved with the highest substitution rate, followed by sub-lineage 3.5. The estimated divergence times of the main PRRSV sub-lineages were as follows: 2001.7 [95% HPD (2000.5, 2003.9)] for sub-lineage 1.5, 1979.5 [95% HPD (1976.3, 1980.2)] for sub-lineage 1.8, 1971.4 [95% HPD (1969.1, 1972.6)] for sub-lineage 3.5, and 1844.6 [95% HPD (1842.3, 1850.8)] for sub-lineage 8.7 (Supplementary Figure S3B).

### 3.3 Extensive inter-lineage recombination

**Figure 6:**
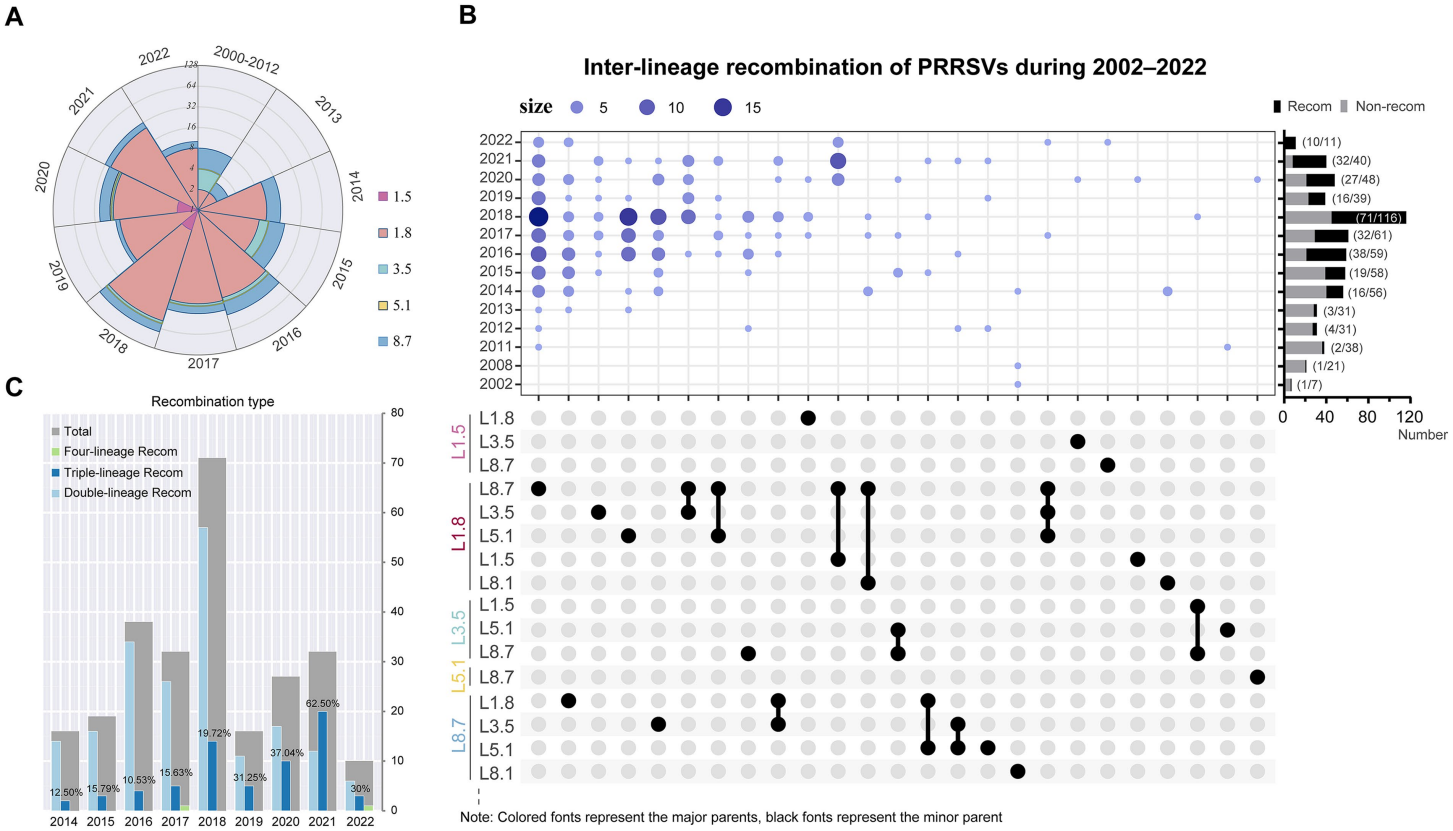
Recombination events drive extensive diversification in PRRSV-2. **(A)** Quantities of major parental strain contributors in recombinants from 2000 to 2022. **(B)** PRRSV-2 recombination landscape. (Top) Bubble chart illustrated the annual distribution of inter-lineage recombination patterns. The rightmost bar chart displayed the annual proportions of recombination events, and the *y*-axis maps to the year, whereas the *x*-axis denotes the count of strains. In the Upset plot, major parents were marked in color, while corresponding minor parents were indicated in black. **(C)** Changes in the inter-lineage recombination frequency of PRRSV-2. The chart annotated the proportion of triple recombinants.

Utilizing integrated outputs from RDP5, Simplot, and VirusRecom program, we identified 273 unique recombination events in the PRRSV dataset (Supplementary Figure S4). In-depth reconstructions around potential breakpoint clusters within these selected sequences could further elucidate the origins and evolutionary trajectories of naturally circulating domestic PRRSV-2 recombinants. Few recombination events occurred prior to 2013. Since 2014, sub-lineage 1.8 has served as the major parent in the majority of recombinant strains (174/273, 63.7%), achieving an almost overwhelming dominance in genetic contribution (Figure 6A; Supplementary Table S2). The most frequent inter-lineage recombination pattern featured mosaics with sub-lineage 1.8 (major parent) + sub-lineage 8.7 (minor parent), followed by those with sub-lineage 8.7 (major parent) + sub-lineage 1.8 (minor parent) (Figure 6B). The recombination landscape has grown increasingly complex, particularly in 2021, where triple-recombinants constituted 62.50%, with the most pronounced pattern featuring sub-lineage 1.8 (major parent) + sub-lineage 8.7 (minor parent) + sub-lineage 1.5 (minor parent) (Figure 6C). Moreover, significant differences existed in recombination hotspots between the two dominant viral sub-lineages. Recombination breakpoints in sub-lineage 1.8 strains were primarily located in regions spanning 0–1,700 nucleotides (5′UTR to NSP1), 5,700–8,400 nucleotides (NSP5–NSP9), and 12,500–13,200 nucleotides (ORF2–ORF4) (Supplementary Figure S5A). In contrast, high-frequency recombination regions in sub-lineage 8.7 strains were mainly distributed within the 1,900–3,600 nucleotides (NSP2) and 12,300–14,900 nucleotides (ORF2 to ORF7) ranges (Supplementary Figure S5B).

Using the set of breakpoints inferred by RDP5, we found significant clustering associated with hot- or cold spots of recombination and compared results from two tests—BDT and RRT (Supplementary Figure S6). Intriguingly, a considerable degree of overlap has been observed between breakpoint hotspot locations and TRS-regulatory motifs among PRRSV-2 isolates (Supplementary Figure S6). This tendency highlights that certain genomic areas may be more vulnerable to template switching during replication. We further explored the configurations of TRS across various PRRSV sub-lineages. TRS-proximal sequences appear relatively conserved within sub-lineage 8.7 strains yet heterogeneous within sub-lineage 1.8 strains (Supplementary Figure S7). These variations in TRS single nucleotide sequences may, to some extent, underlie differential recombination dynamics between distinct viral sub-lineages.

### 3.4 Glycosylation patterns and functional domain analysis of GP5

**Figure 7:**
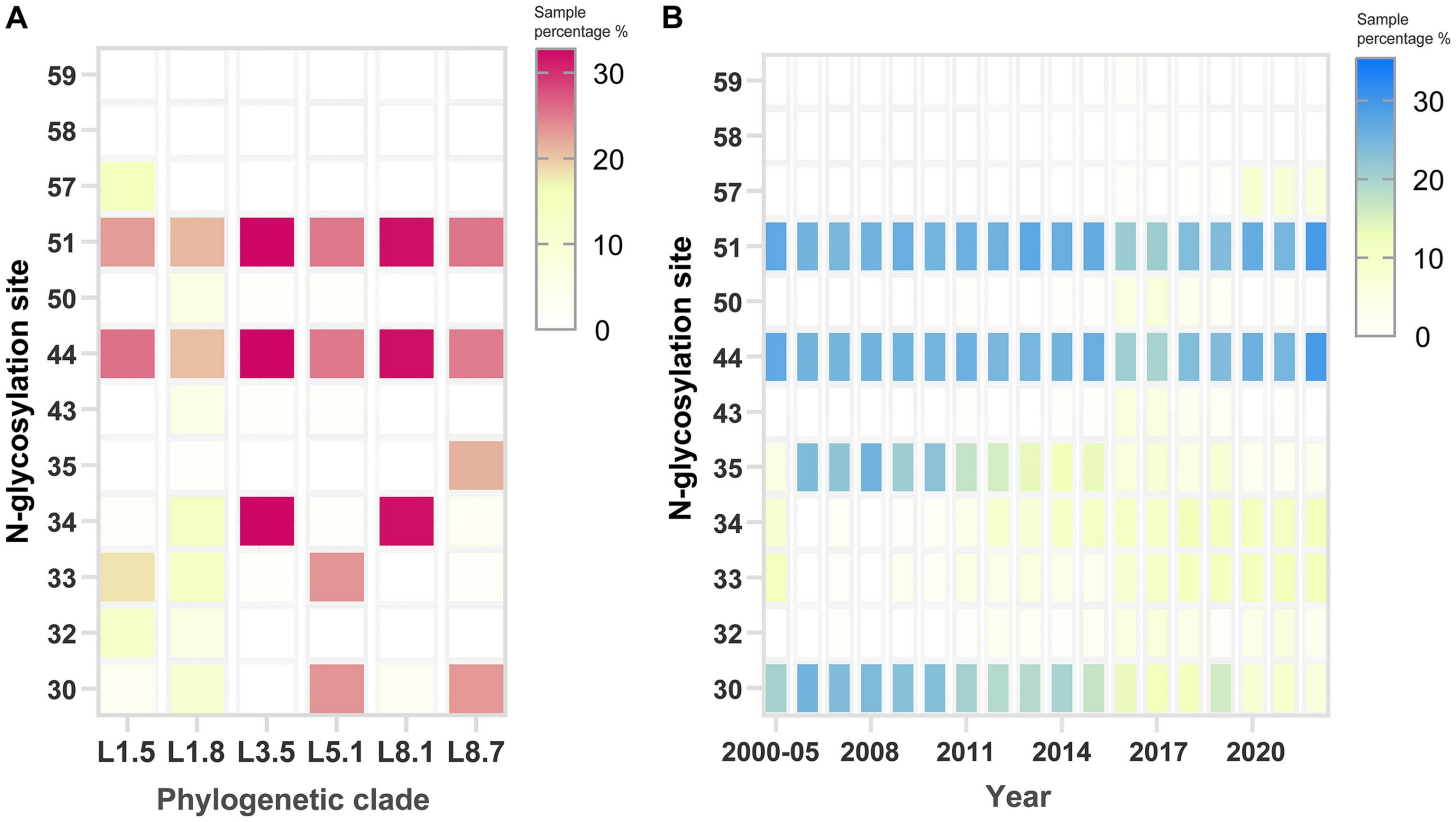
Identification of potential N-glycosylation patterns on the GP5 protein of PRRSV-2. Analysis of N-glycosylation site distribution across **(A)** phylogenetic clades and **(B)** sampling years. The rightmost scoring bar per panel shows the percentage of N-glycosylation sites within the strains. The default threshold value was configured to 0.5 on the specified web server.

N-glycosylation analysis across PRRSV sub-lineages revealed considerable variability in site occupancy patterns (Supplementary Table S1). Although the glycosylation profiles are not strictly lineage-defining, some conserved patterns are still observed. Specifically, the majority of sub-lineage 5.1 strains displayed occupancy at N30, N33, N44, and N51, whereas sub-lineage 8.7 predominantly utilized N30, N35, N44, and N51, and sub-lineage 3.5 chiefly employed N34, N44, and N51. Marked polymorphism in N-glycosylation was most pronounced for sub-lineages 1.5 and 1.8, with almost no fixed pattern observed (Figure 7A). Residues N44 and N51 were largely conserved across various sub-lineages. Notably, N57 emerged in recent years, with its usage exclusive to sub-lineage 1.5 strains. Conversely, residues 30 and 35 showed a gradual loss of glycosylation potential over the timeline (Figure 7B). Collectively, these glycosylation pattern fluctuations coincided with the historical PRRSV epidemics in China.

Comparison of the primary neutralizing epitope (S37HLQLIYNL45, Epitope B) among known antigenic determinants revealed distinct amino acid substitutions across viral sub-lineages. Residue 39 was particularly polymorphic (Supplementary Figure S8A). As enumerated in Supplementary Figure S8B, all sub-lineage 8.7 strains contained the L39I mutation, whereas sub-lineage 8.1 viruses harbored the L39F mutation. Sub-lineage 3.5 strains uniquely displayed mutations of H38Y alongside L39S. Examination of the decoy epitope (27A/VLVN30, Epitope A) further revealed lineage-specific differences, with the A29V mutation occurring in nearly all sub-lineages 1.5, 1.8, and 3.5 isolates (Supplementary Figure S8B). Furthermore, a N30S mutation was identified in sub-lineage 3.5 isolates.

## 4 Discussion

Retrospective studies showed that the first PRRSV strain (CH-1a) was isolated from an aborted piglet in mainland China in 1996 (Guo et al., 2018). Subsequent multiple virus importation events propelled the emergence and spread of new outbreaks around the country (Zhou et al., 2015). The extensive genetic and antigenic heterogeneity among PRRSV variants results in poor cross-neutralization, presenting a major barrier to effective immunization (Nan et al., 2017). Competitive shifts in lineage dominance were observed in our investigation, from lineage 8 to lineage 1. Several hypotheses have been proposed to explain the replacement of PRRSV clade/lineage (Paploski et al., 2021). Upon initial emergence in swine populations, the genetic diversity of ancestral viral strains is typically low. As novel variants emerge with fitness advantages, they are able to expand then supersede predecessors through competitive transmission dynamics. Similar observations were independently made in the United States, where phylogenetic analyses revealed the sequential dominance of distinct PRRSV sub-lineages over time (Paploski et al., 2019). On average, newly dominant sub-lineages emerged and reached peak population sizes every 4.5 years (Paploski et al., 2021). Undoubtedly, elucidating the drivers of shifts in lineage dominance will prove critical for curbing endemic transmission and mitigating future pandemic risks.

Lineage 1 isolates displayed more extensive signs of recombination as well as significant variations in virulence and pathogenicity phenotypes than other lineages (Liu et al., 2022; Wu et al., 2024). However, the consequences of recombination on viral fitness remain unknown. Partial lineage 1 recombinants (FJ1402, JL580 etc.) exhibited high pathogenicity comparable to HP-PRRSV (Guo et al., 2018). We previously determined that lineage 1 recombinants (XM-2020, YC-2020) displayed enhanced replicative advantage in pulmonary alveolar macrophages (PAM) cells (Zhang et al., 2021; Wang et al., 2022). Recombinants between divergent viral lineages have the capacity to unite potential advantageous phenotypic properties, conferring more evolutionary options than would be available to them by mutation alone. Evidence suggested an inverse correlation between recombination efficiency and fidelity for viral RNA-dependent RNA polymerase (RdRp), known low-fidelity RdRp variants tended to exhibit increased recombination frequencies (Campagnola et al., 2022). Moreover, our research also revealed contrasting recombination and evolutionary dynamics between virus lineages circulating in China, with some identifiable patterns. Specifically, we confirmed the non-randomness of recombination breakpoints, located high-frequency recombination hotspots within the two dominant viral sub-lineages 1.8 and 8.7, and further detected associations between breakpoint clusters and TRS sites. Concordant with observations in previous studies on coronavirus recombination, sequence features such as TRSs have been implicated as major determinants of template switching-mediated recombination events (Yang et al., 2021; De Klerk et al., 2022). AU-rich regions facilitate more efficient recombination and template switching (D’Souza et al., 2021). Currently, the presence of recombinant-resistant PEDV RMT mutant has been highlighted by remodeling the transcription regulatory sequence-core sequences (TRS-CSs) of PEDV using reverse genetics (Niu et al., 2023). According to our results, lineage 1 exhibited a high recombination propensity toward genomic regions involved in viral replication or protein processing, with such rearrangements may be linked to enhanced replicative fitness and cellular tropism. This research better quantifies the contribution of recombination in shaping PRRSV-2 evolution and genetic diversity, underscoring the necessity of monitoring recombinant PRRSV strains. Elucidating the mechanisms underlying the increased recombination potential may reveal new antiviral targets for suppressing their evolutionary capacity.

Effective population sizes have been observed to differ across viral sub-lineages, with episodic expansions and contractions in genetic diversity occurring periodically within circulating lineages. These lineage-specific dynamics may be driven by a combination of factors, including the emergence of antigenic variants that temporally evade accumulation of population immunity. The increased N-glycosylation sites and shifting glycosylation patterns provide key evidence for adaptive viral evolution in response to host humoral immunity (Paploski et al., 2022). The putative N-gly sites in field isolates of PRRSV sub-lineages 1.5 and 1.8 demonstrated highly diverse and variable patterns, while being conserved in other sub-lineages. Early data suggested that PRRSV immune evasion pathways involve modifications to glycan shields; it has been established that the loss of glycan residues in N34 and N51 enhanced the immunogenicity of the proximal neutralizing epitope (Ansari et al., 2006). Certain residues within these hypervariable regions have shown to be under positive selection pressure, and variants antigenically distinct from previously circulating sub-lineages may possess adaptive advantages (Rupasinghe et al., 2022). In summary, these findings elucidate the macroevolutionary patterns driving shifting PRRSV dominance in China; thus laying the foundation for developing prevention and containment strategies against PRRSV.

## Data availability statement

The datasets presented in this study can be found in online repositories. The names of the repository/repositories and accession number(s) can be found in the article/Supplementary material.

## Author contributions

RZ: Investigation, Methodology, Software, Writing – original draft, Writing – review & editing. HL: Conceptualization, Investigation, Software, Supervision, Writing – original draft. HX: Data curation, Methodology, Writing – original draft. XH: Methodology, Software, Supervision, Writing – original draft. LZ: Supervision, Visualization, Writing – original draft. AC: Project administration, Resources, Writing – review & editing. BZ: Methodology, Writing – review & editing. YZ: Data curation, Supervision, Writing – review & editing. XW: Conceptualization, Funding acquisition, Project administration, Resources, Supervision, Writing – review & editing.

## Footnotes

^1^ https://www.geneious.com

^2^ http://tree.bio.ed.ac.uk/software/figtree/

^3^ https://services.healthtech.dtu.dk/services/NetNGlyc-1.0/
