# Supplementary figures and images for: Comparing the molecular evolution and recombination patterns of predominant PRRSV-2 lineages co-circulating in China
Source: Front Microbiol. 2024 Apr 26;15:1398470. doi: 10.3389/fmicb.2024.1398470 (PMC11088243; doi:10.3389/fmicb.2024.1398470)

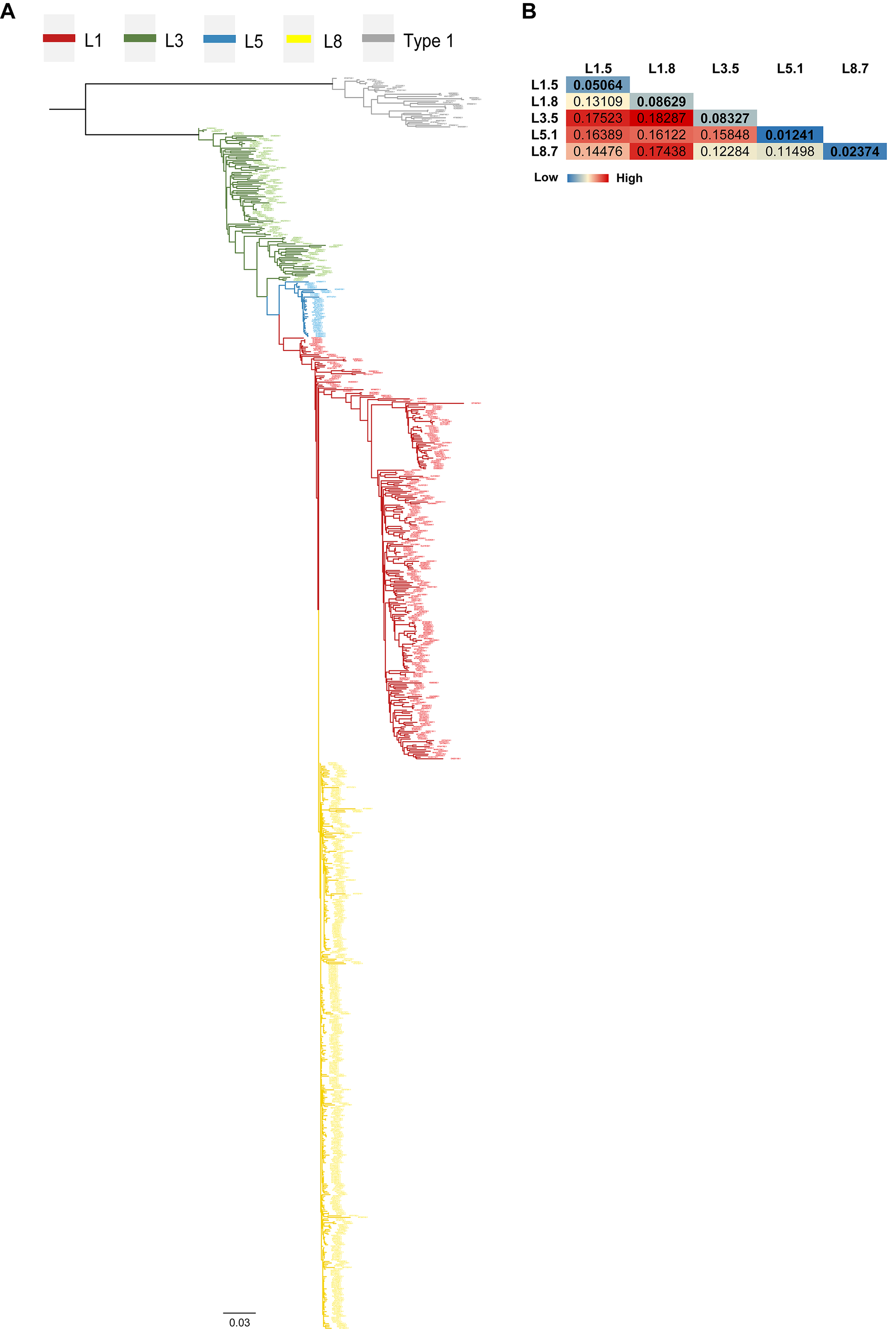

Supplement: Supplementary file 1 [file Image_1.TIF]

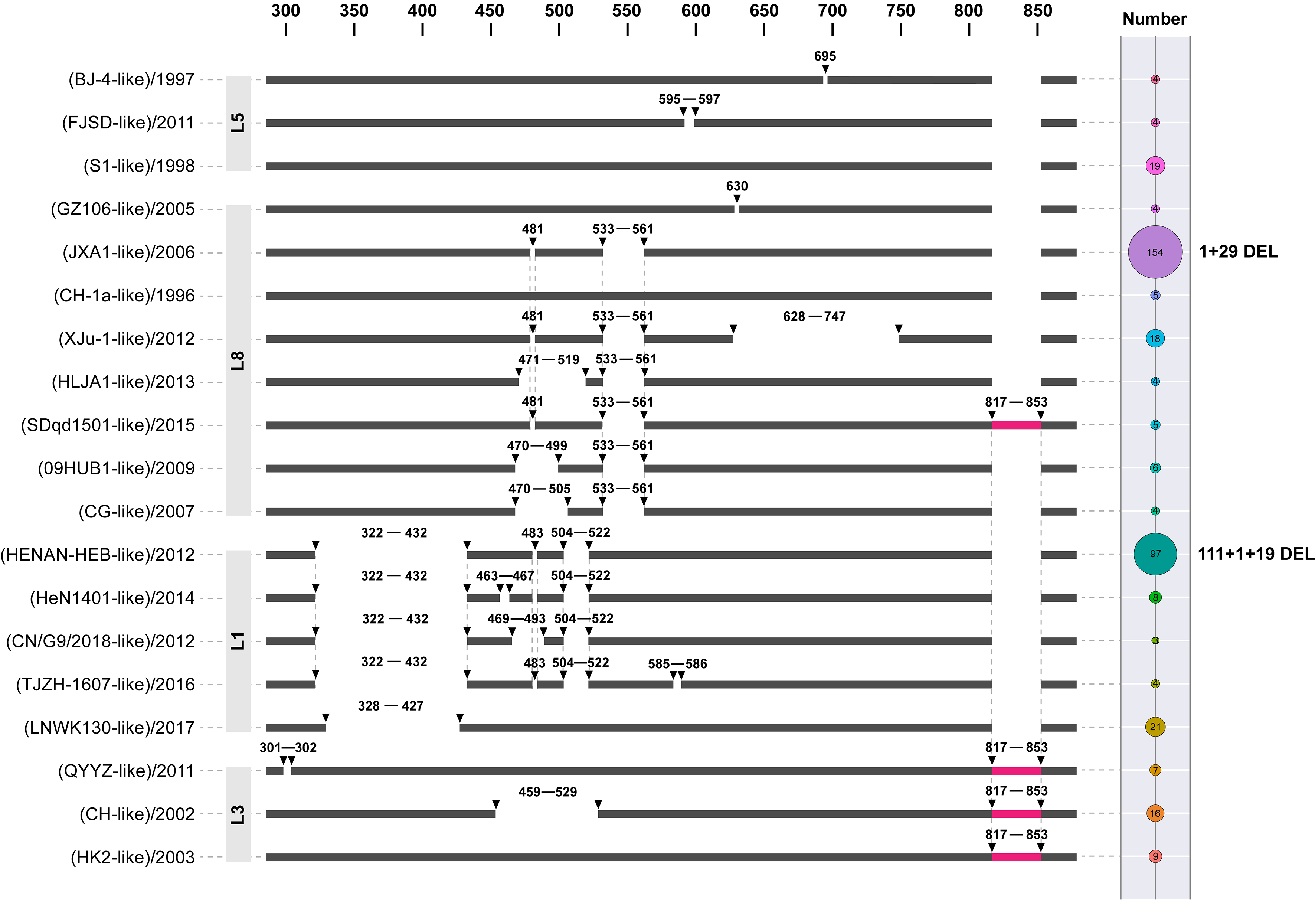

Supplement: Supplementary file 2 [file Image_2.TIF]

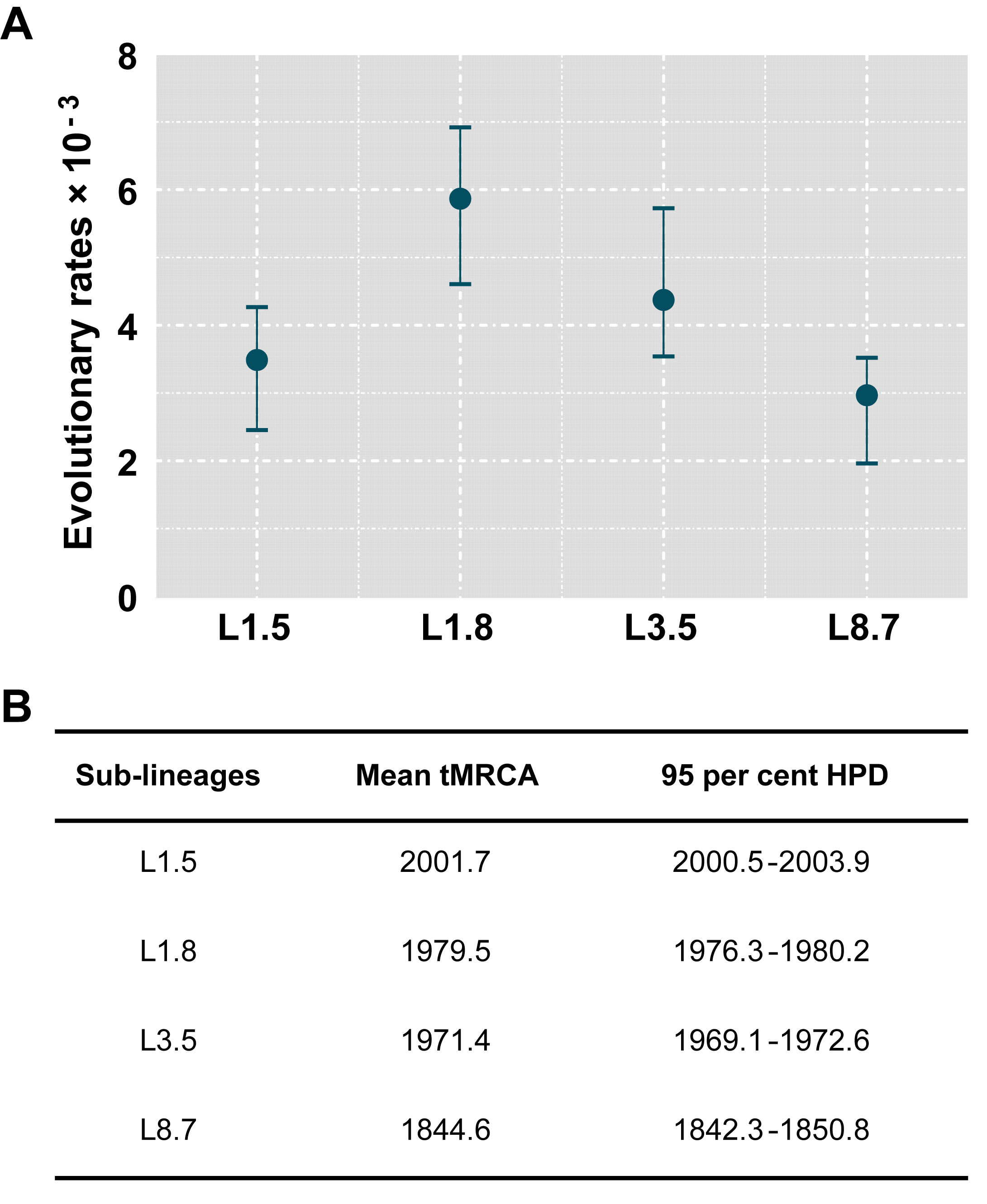

Supplement: Supplementary file 3 [file Image_3.TIF]

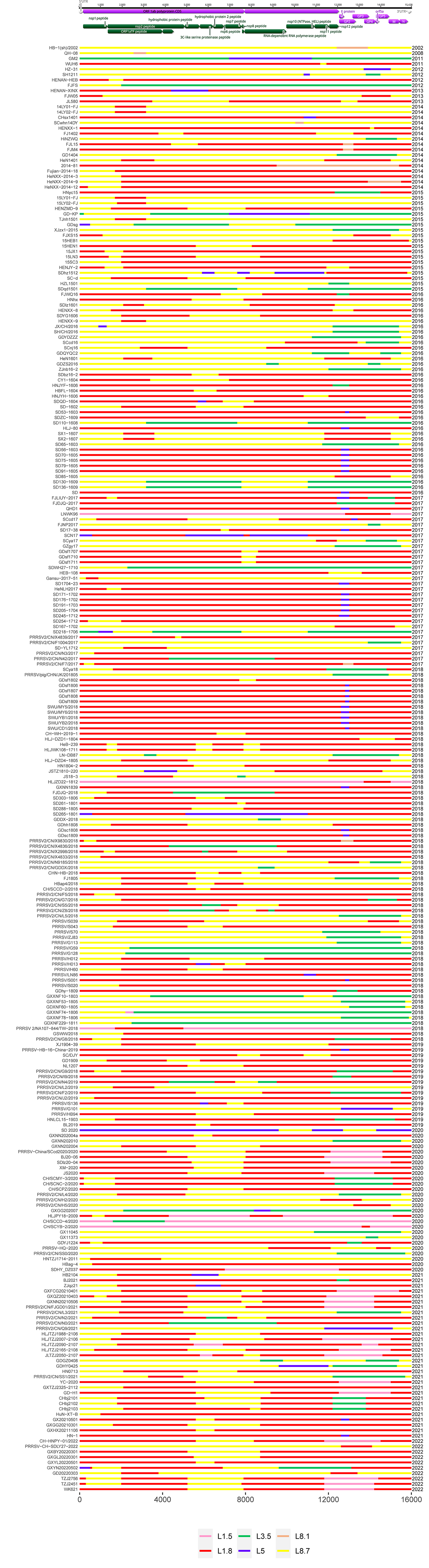

Supplement: Supplementary file 4 [file Image_4.TIF]

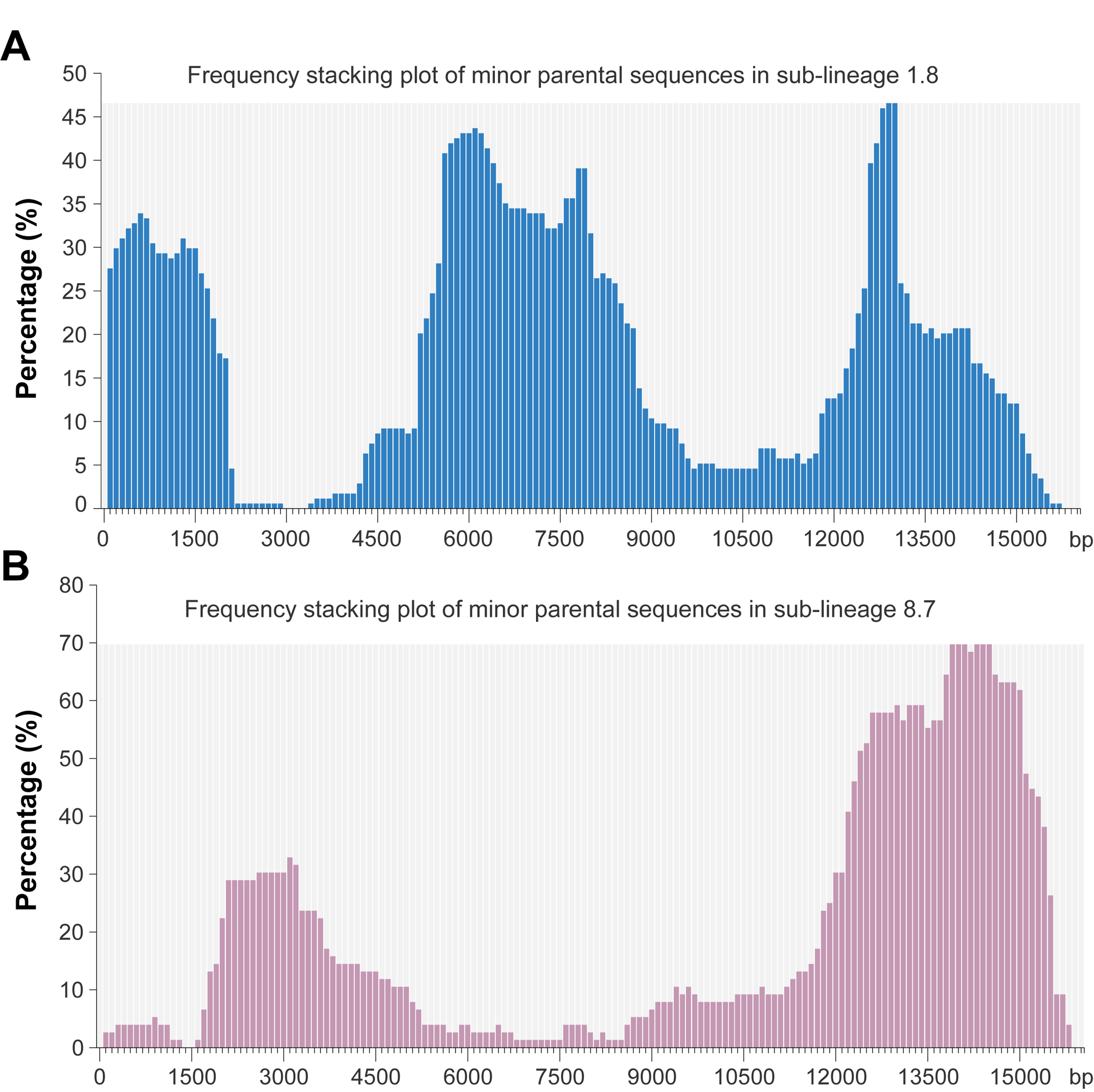

Supplement: Supplementary file 5 [file Image_5.TIF]

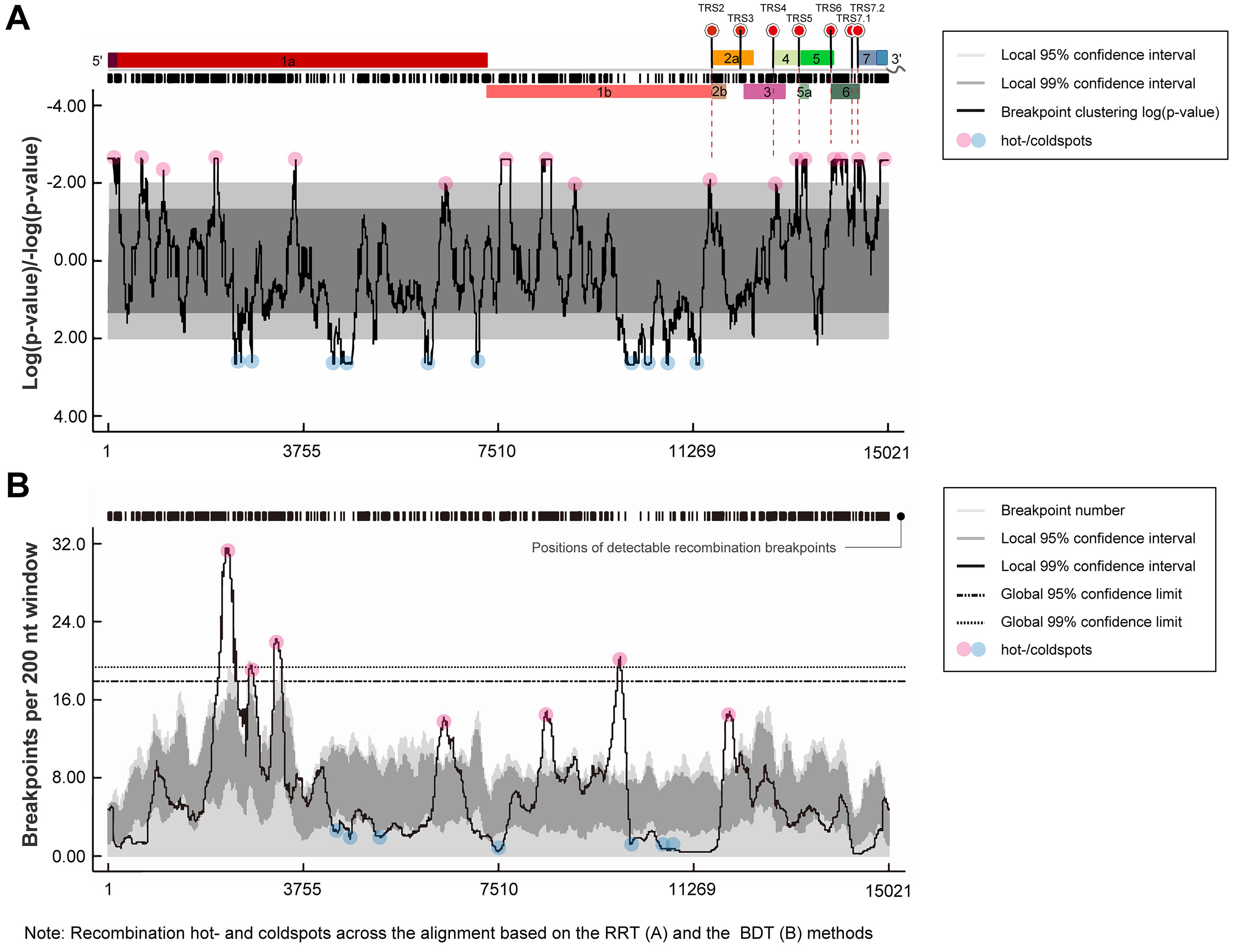

Supplement: Supplementary file 6 [file Image_6.TIF]

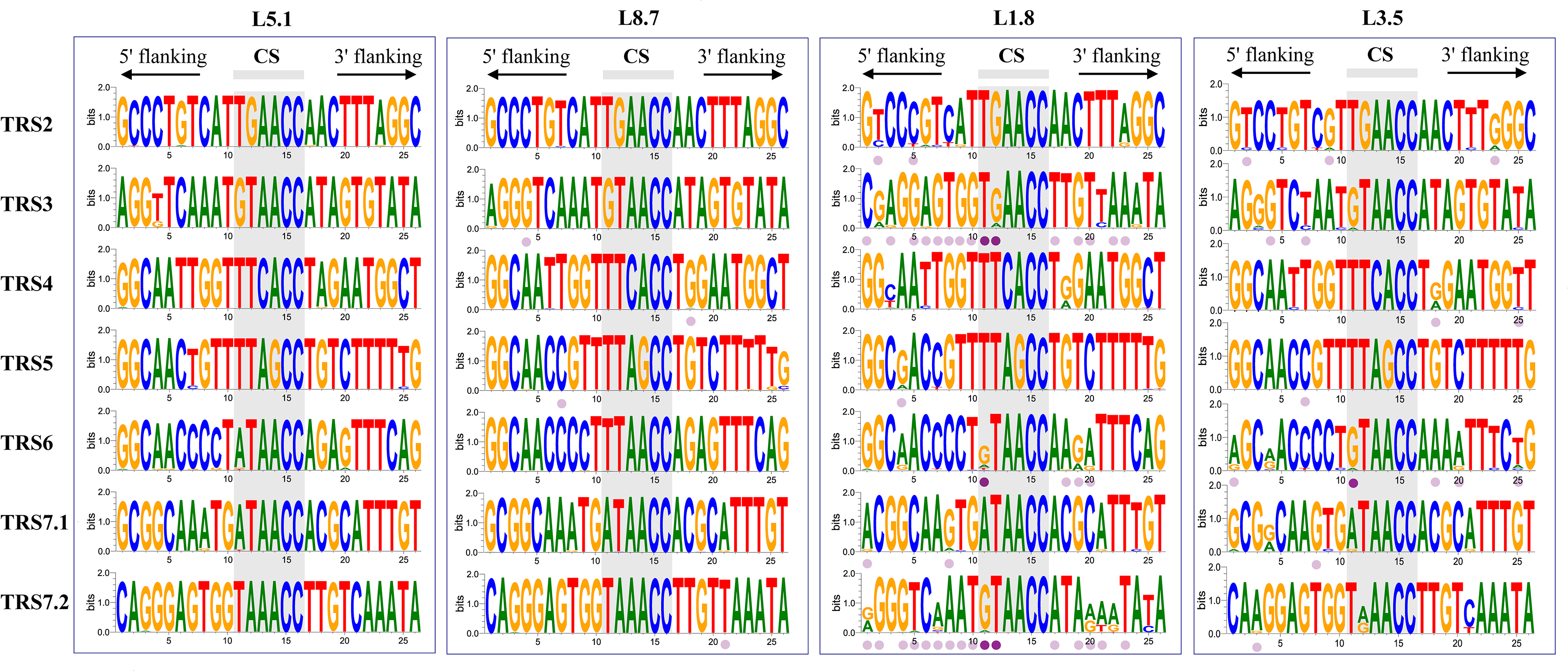

Supplement: Supplementary file 7 [file Image_7.TIF]

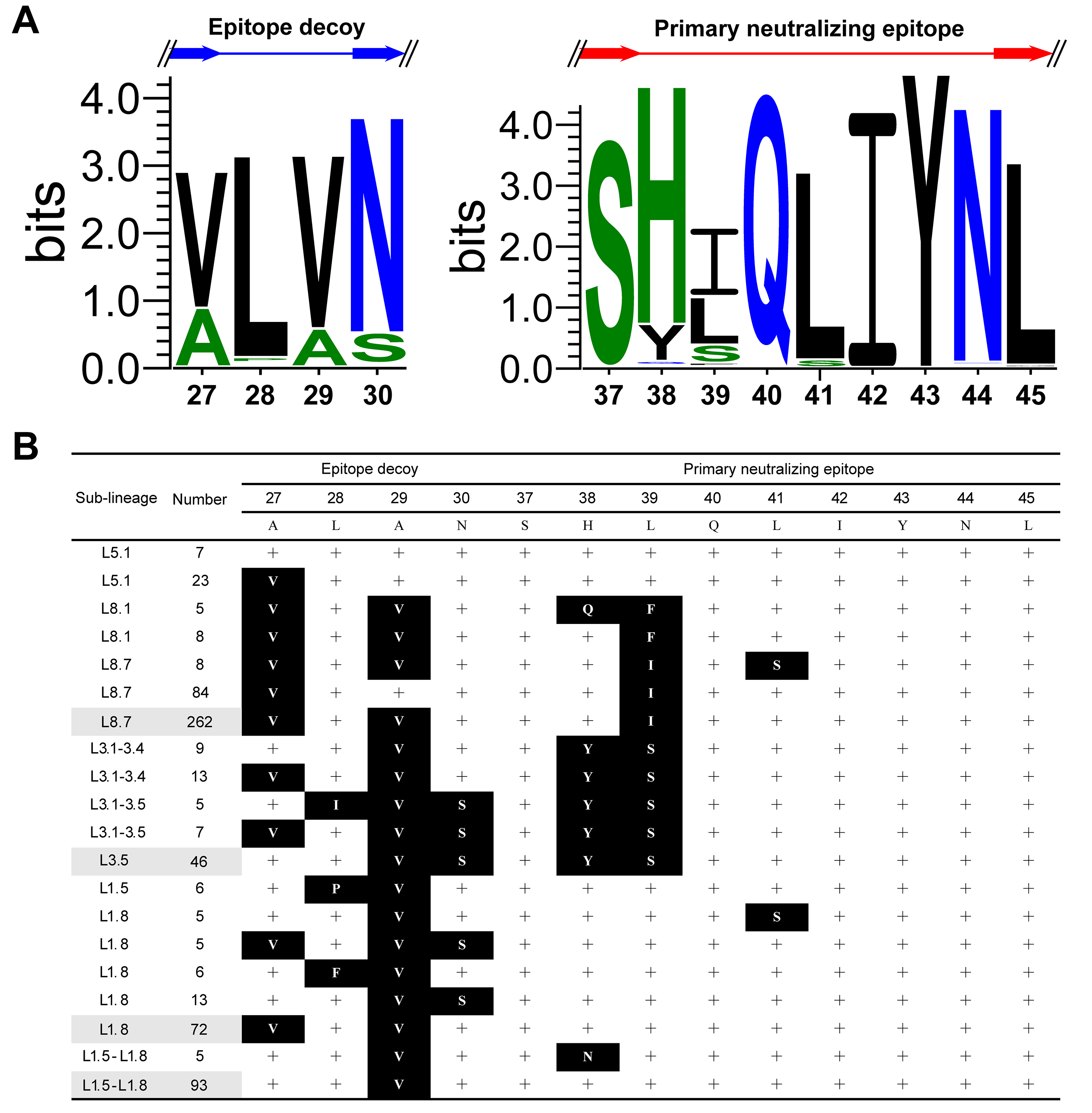

Supplement: Supplementary file 8 [file Image_8.TIF]
