# Supplementary material for: Comparing the molecular evolution and recombination patterns of predominant PRRSV-2 lineages co-circulating in China
Source: Front Microbiol. 2024 Apr 26;15:1398470. doi: 10.3389/fmicb.2024.1398470 (PMC11088243; doi:10.3389/fmicb.2024.1398470)
Supplement: Supplementary file 11 [file Table_3.DOCX]

**Figure captions in supplementary materials:**

**Supplementary Figure S1.** **Analyses of phylogenetic and genetic distance were conducted based on the ORF5 sequence dataset.** (**A**) Maximum likelihood phylogeny with GTR+F+I+G4 model and 1,000-replicate ultrafast bootstrap was constructed. (**B**) Mean genetic distance between and within sub-lineages was measured using the p-distance model.

**Supplementary Figure S2.** **Insertion and deletion patterns of NSP2 in Chinese PRRSV-2 strains** **(Supported by ≥3 Isolates).** Arrows and numerals denote the indel positions within NSP2, referencing the VR-2332 strain coordinates. The text annotation on the left indicates the representative strain in which this pattern was first identified. Right vertical axis represents the number of strains supporting this pattern.

**Supplementary Figure S3. The mean evolutionary rates and tMRCA for different PRRSV-2 sub-lineages.** (**A**) Nucleotide substitution rates per site per year across each sub-lineage. (**B**) Summary of the most recent common ancestors of viral sub-lineages.

**Supplementary Figure S4.** **Unique inter-lineage recombination analysis among PRRSV-2 isolates.** Strain names are listed on the far left, with their corresponding collection date on the far right. Distinct colors represent the corresponding viral sub-lineages of the major parental strains; alterations in color denote replacement of the segment by a minor parental strain. The positions corresponded to the PRRSV VR-2332 strain.

**Supplementary Figure S5.** **The proportions of recombinant genomes featuring minor parent sequences in sub-lineages 8.7 and 1.8 strains.** The x-axis indicates the PRRSV genomic position, and the y-axis quantifies the breakpoint probability distributions of recombination events, utilizing sliding windows of 100 nucleotide bases each.

**Supplementary Figure S6.** **Breakpoint clusters inferred by RDP5.** Distribution of recombination hotspots and coldspots across the alignment based on RRT (**A**) and BDT (**B**) methods. Red dots above represent recombination hotspots, while the blue dots below indicate coldspots. The corresponding TRS genomic schematic is annotated with vertical black lines on the chart.

**Supplementary Figure S7.** **Heterogeneity of TRSs in subgenomic RNAs across various PRRSV sub-lineages.** Core sequences (CS) are indicated inside gray boxes. Single nucleotide variations are annotated with purple dots at the bottom, relative to the VR-2332 genome.

**Supplementary Figure S8.** **Amino acid analysis of GP5 protein.** (**A**) Web-logos highlight the variability within the decoy epitope and primary neutralizing epitope. (**B**) Lineage-specific differences in epitopes. ''+'' represents the identical amino acids.
